# Exploring needs and perceptions about telemental healthcare among Veterans with spinal cord injuries, a qualitative study

**DOI:** 10.1007/s10865-026-00668-0

**Published:** 2026-04-20

**Authors:** Hilary N. Touchett, Kelley Arredondo, Brenda Salgado, Trenton M. Haltom, Stephanie C. Day, Andreas Weyland, Larissa Grigoryan, Jan Lindsay, Drew A. Helmer, Felicia Skelton

**Affiliations:** 1 Center for Innovations in Quality, Effectiveness and Safety (IQuESt), Michael E. DeBakey VA Medical Center, Houston, TX USA; 2 VHA Office of Rural Health’s Veterans Resource Center, Salt Lake City, United States of America; 3 Department of Medicine, Baylor College of Medicine, Houston, TX USA; 4 South Central Mental Illness Research, Education, Clinical Center, a Virtual Center, Houston, USA; 5 VHA Office of Rural Health’s Veterans Resource Center, White River Junction, VT USA; 6 Department of Psychiatry and Behavioral Sciences, Baylor College of Medicine, Houston, TX USA; 7 School of Medicine, Baylor College of Medicine, Houston, TX USA; 8 Department of Family Medicine, Baylor College of Medicine, Houston, TX USA; 9 Department of Physical Medicine and Rehabilitation, Baylor College of Medicine, Houston, TX USA

**Keywords:** Spinal cord injuries (SCI), Mental health (MH), Access to care, Telehealth, Qualitative, Veteran

## Abstract

Mental health (MH) is essential for self-efficacy and self-care, both of which affect health outcomes and care costs for Veterans with disabilities. Co-occurring mobility impairment and MH disorder are strongly linked to reduced daily function and lower life satisfaction. Veterans with spinal cord injuries (SCI) are a high-priority group for VA and are frequent, high-cost healthcare users. MH disorders are common in Veterans with SCI, but are often underdiagnosed and undertreated due to medical complexity, limited clinician familiarity with post-injury adjustment, and insufficient evidence for effective MH treatments in this population. Telehealth can increase access to MH care but requires supports such as accessible applications, adaptive equipment, and electronic health literacy. This qualitative study presents interview findings on how Veterans with SCI experience and perceive telemental healthcare. We conducted semi-structured interviews with 24 Veterans diagnosed with SCI to learn about their needs, experiences, and perceptions of telemental healthcare. Interviews were thematically analyzed. Participants described diverse experiences with telemental healthcare, with most finding video telehealth helpful for improving access to care. Veterans emphasized three key findings: the value of virtual care in overcoming distance and SCI-related challenges; the need for providers to have comprehensive knowledge of available resources and to deliver disability-competent care; and the importance of flexible telehealth options tailored to individual needs. Four primary themes emerged: (1) Telemental health addresses distance and SCI related Access Challenges, (2) Unintended Consequences Impact Access and Accessibility of Telemental Health, (3) Adaptive Strategies May Help Mitigate Telemental Health Barriers, and (4) Flexible Use of Telehealth Can Match Care Modalities to Individual Needs. Telehealth is an important tool for Veterans with SCI when seeking MH care. However, individualized support is needed to address barriers and facilitate positive experiences, thereby maximizing therapeutic benefit.

## Introduction

Mental health (MH) is key to self-efficacy and self-care, which, in turn, shape chronic disease management, quality of life, and healthcare use among Veterans. Strong self-efficacy supports coping (Zhou et al., 2021), positive health behaviors (Schmutte et al., 2009), treatment adherence (Lee et al., 2021), and fewer rehospitalizations in chronic conditions, including spinal cord injury (SCI; Gassaway et al., 2017). After SCI, individuals must manage complications and prevent secondary conditions. Effective self-care helps reduce rehospitalization due to pressure injuries, medication issues, cardiac events, respiratory infections, UTIs, and kidney injury (Vecin & Gater, 2022). SCI self-management increases reliance on behavioral medicine strategies—problem-solving, symptom monitoring, adherence, and coping—to support ongoing health. Veterans with both mobility impairments and a MH diagnosis report fewer functional days (ie, days where usual duties and activities can be performed) and lower life satisfaction, highlighting the need for integrated physical and MH care (Merikangas et al. 2007; Na & Singh, 2021).

### Spinal cord injury in the Veteran population

**Fig. 1:**
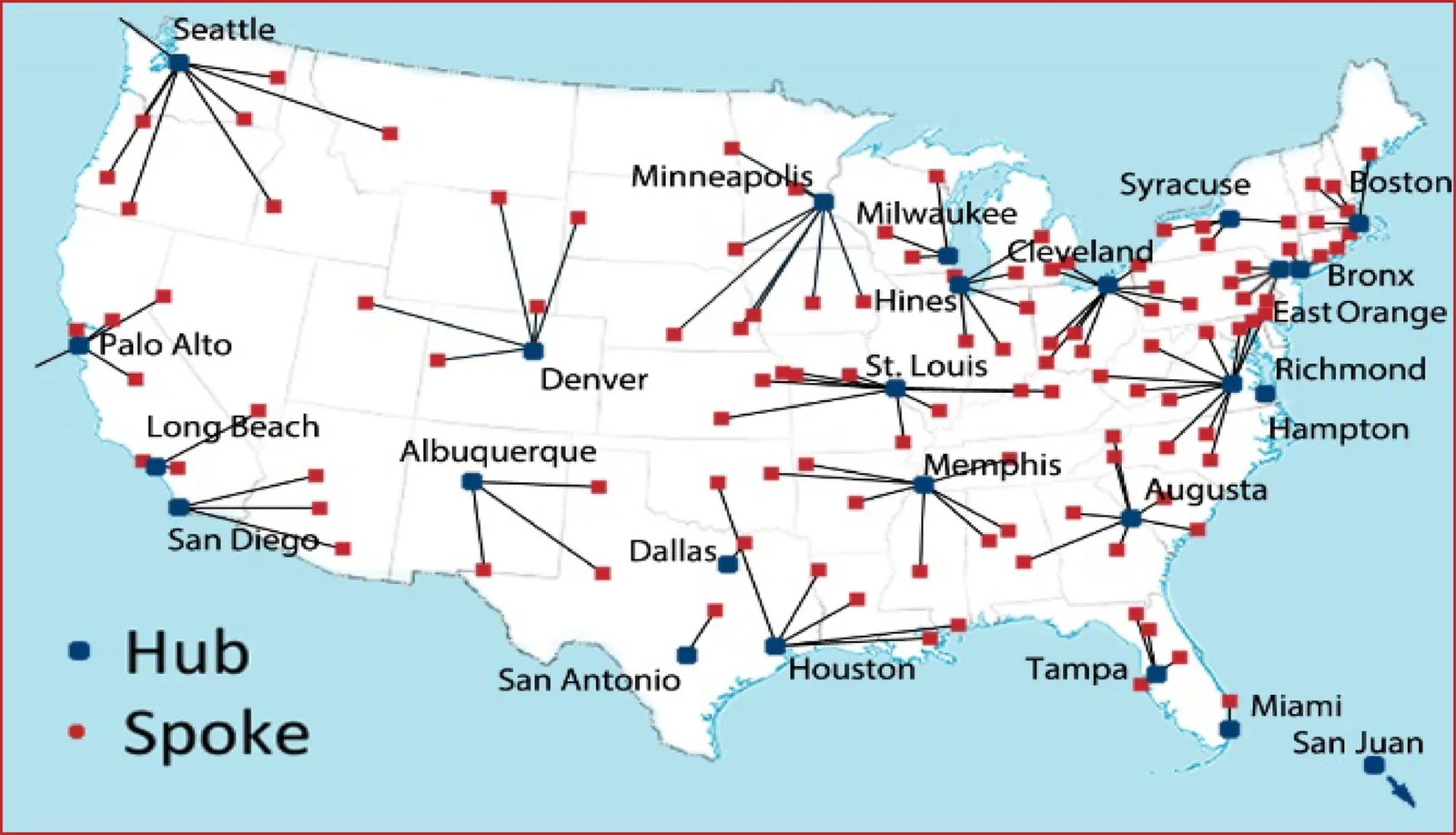
The VA SCI hub and spoke system. The VA SCI system of care is a coordinated national network of facilities

**Fig. 2:**
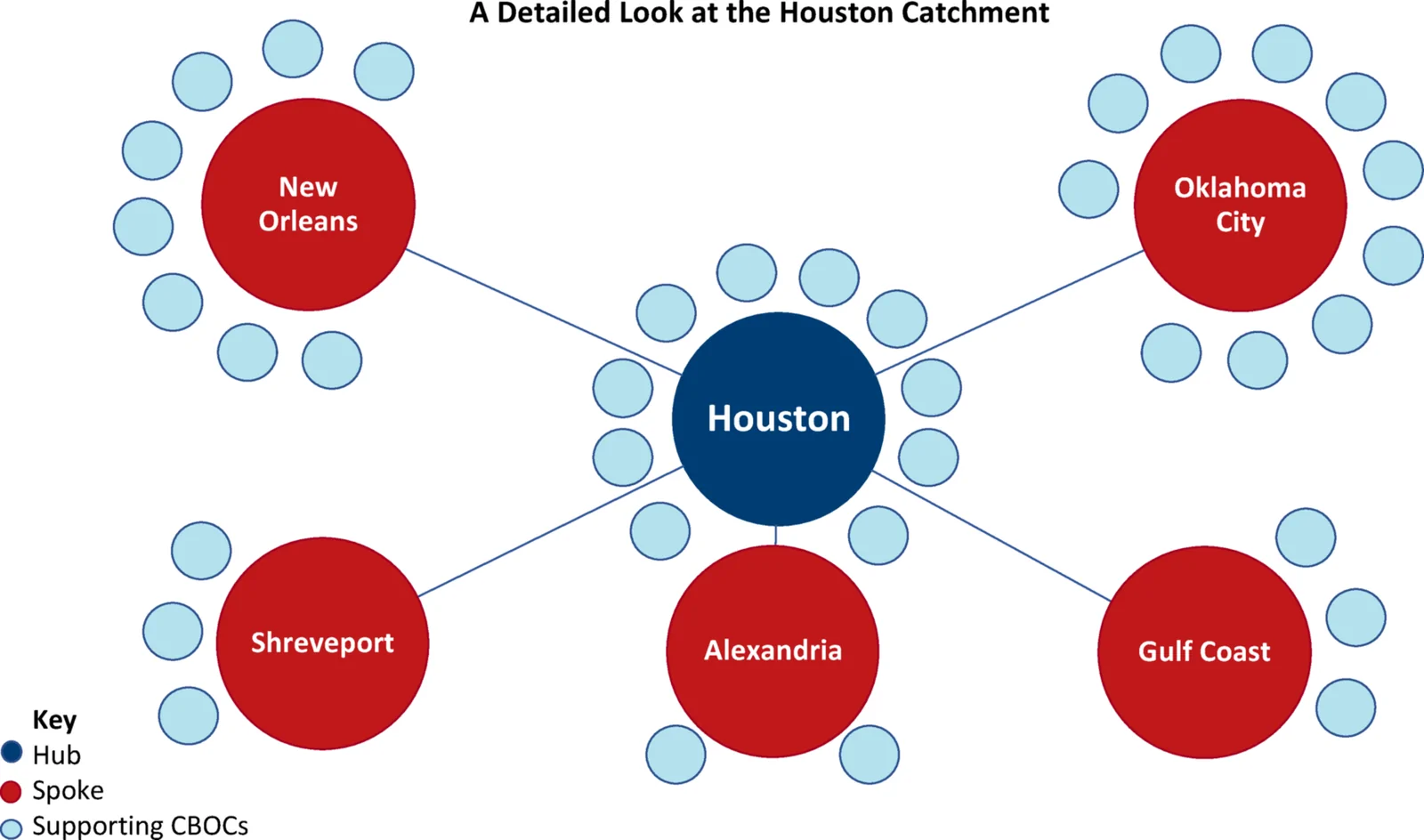
A close up of a SCI catchment. The SCI system is comprised of more than SCI hubs and spoke site medical centers; each VA medical center also supports surrounding community-based outpatient clinics in neighboring towns in this comprehensive network

Within the Department of Veterans Affairs (VA), Veterans with SCI are a high-priority population characterized by complex medical needs, high service utilization, and elevated costs of care (Skelton et al., 2015). Annually, the VA SCI and Disorders (SCI) system of care provides care to nearly 24,000 Veterans with SCI (Daly et al., 2025; Martinez et al., 2022). The VA SCI system of care operates using a "hub and spoke" delivery model designed to efficiently deliver specialized expertise across each catchment (Sippel et al., 2025). This model resembles a wheel, with 25 SCI Centers of Excellence serving as regional hubs, offering advanced clinical capabilities and guidance to their catchment areas, and the affiliated VA medical centers and outpatient clinics functioning as spokes. Figure 1 depicts the national system of care with primary and secondary care layers, whereas Fig. 2 provides a closer look at the tertiary care level, including community-based outpatient clinics that feed into this system.

These SCI hubs employ multidisciplinary patient-aligned care teams comprising physicians, advanced practice providers, nurses, psychologists, social workers, case managers, pharmacists, physical, occupational, and speech therapists, as well as telehealth coordinators. SCI care within the VA is guided by VA directive 1176 (*VHA Directive 1176(2)*, 2020), which directs Veterans to receive a comprehensive annual assessment by the SCI team that includes a full medical evaluation, relevant laboratory and radiographic testing, along with functional and psychosocial assessments. These annual exams are used to set the Veteran’s plan of care, which is then carried out by the spoke site facilities closest to the Veteran’s home, meaning the ongoing care team may have varying levels of SCI training.

### Mental health and SCI

MH disorders are common in Veterans with SCI, showing a need for SCI-focused MH care. Nearly half have depression (Hatch et al., 2021), a third have anxiety (Bombardier et al., 2021; Le & Dorstyn, 2016), and a fourth have PTSD (McDonald et al., 2018). People with SCI are 3–5 times more likely to die by suicide than those without impairment (Cao et al., 2014; Hartkopp et al., 1998). Yet, MH and substance use disorders are often underdiagnosed and undertreated in people with SCI. This is due to medical complexity, limited provider familiarity with adjustment after catastrophic injury, and not enough evidence on effective MH treatments for SCI (Bombardier et al., 2021; Fann et al., 2011; Le & Dorstyn, 2016; Martinez et al., 2022; McDonald et al., 2018).

### Telehealth for MH care among Veterans with SCI

When addressing MH needs for Veterans with SCI, consider how various intersecting factors complicate seeking and receiving MH. Veterans with MH conditions face added barriers, such as privacy concerns, lack of trust in VA, and difficulty navigating the system (Cheney et al., 2018; Fischer et al., 2016). For people with SCI, these challenges worsen due to disability-related barriers, including inaccessible transport, limited wheelchair-accessible provider offices, and reliance on others for travel or help (Clemente et al., 2022; N. Hamilton et al., 2022; R. Hamilton et al., 2017; Matin et al., 2021).

Video telehealth for mental health (telemental health) improves access and continuity of care for Veterans with SCI. VA uses VA Video Connect (VVC), a secure, VA-created video telehealth platform with end-to-end encryption (Myers et al., 2021). Home-based telemental health can lower access barriers by reducing travel burden (Jacobs et al., 2019). It can improve therapeutic alliance over audio-only methods (Chen et al., 2021) and support care engagement (Perry et al., 2020). However, provider knowledge, misperceptions about veterans’ technology use, and disability-specific barriers may limit effectiveness (Clemente et al., 2022; Matin et al., 2021; Touchett et al., 2021).

To ensure equitable and effective MH care, telemental health should address both access and accessibility. These terms overlap but are different, and each needs unique solutions. In this study, access means the Veteran has the theoretical ability to use telemental health, such as having a good internet connection or an internet-enabled device (Annaswamy et al., 2020; Kobeissi & Manning, 2025; Valdez et al., 2021). Accessibility means the application is usable and easy to use.

*Study Aim* This qualitative study explored the experiences and needs of Veterans with SCI regarding telemental health. Findings aim to inform strategies to improve access and quality of MH care for Veterans with SCI.

## Methods

We conducted semi-structured interviews with Veterans with SCI to understand their needs, experiences, and perceptions of telemental health. Using purposive sampling, Veterans could participate in interviews if they were in the SCI registry, assigned to the (blinded) SCI hub, attended an Annual Exam in the past year, spoke English, and could provide consent. We recruited Veterans by sending opt-out letters to all patients in the (blinded) catchment area. We started recruiting at the hub site and moved to each spoke site. We called patients to check interest, completed consent, and scheduled interviews.

### Data collection

Interviews were conducted between April 2021 and July 2022, during the later stages of the COVID-19 pandemic. We developed two interview guides based on clinical issues identified at the time—one for those who had used any telehealth in the past and those who had not—to elicit experiences, perceptions, and needs surrounding telemental health as well as rationales for why telemental health was not used (e.g., preference, lack of availability). Veterans who had used telehealth before were asked about: telehealth context (e.g., platforms used, where conducted, technology familiarity), overall experience and perceptions, barriers encountered, adaptive equipment and other support needed, recommendations for or against telemental health, and how to make telemental health easier to use for other Veterans with SCI. Veterans who had not used telehealth were asked: why they never used telehealth (e.g. preference for another format of care, never been offered, lack of equipment/support), what they have heard about telehealth, experiences with MH care in the VA, and considerations for telemental health (e.g., whether they would consider using telemental health, what would be needed to facilitate a visit).

Data collection ended when all eligible participants in the (Blinded) SCI catchment (see Fig. 2) had been contacted. Two interviewers conducted all interviews one-on-one over the phone (audio only) using the Zoom call-out function (telephone) to allow for recording. Calls lasted 36 min on average (13–60 min). Interviewers had no prior relationship with Veterans prior to interviews. Interviewers were comprised of a PhD-prepared researcher (1st author) and a Master’s-prepared research coordinator with experience and formal training in qualitative research. They were assisted by a Bachelor’s-prepared research coordinator who was trained to conduct interviews. Interviews were audio-recorded and later transcribed verbatim by the VA Centralized Transcription Services Program. Veterans were compensated $40 in VA Canteen Vouchers, which can be redeemed in VA cafeterias and retail stores for completing interviews.

### Analysis

Using thematic analysis, four analysts (1st, 3rd, 4th, and 5th authors) iteratively coded transcripts using ATLAS.ti, a qualitative data analysis software (ATLAS.ti Scientific Software Development GmbH, 2025). Analysts consisted of three PhD-prepared researchers with experience and formal training in qualitative methods, and one bachelor’s-prepared research coordinator who was trained by the team in qualitative analysis methods. After double-coding 5 transcripts, coders drafted a codebook inductively through iterative discussions until all coders agreed that the final codebook accurately captured the data. All transcripts were analyzed by at least two coders, with disagreements resolved through discussion by all coders. Codes were then organized into themes and subthemes presented in the results.

### Ethical approval

This study was approved by the (blinded) Institutional Review Board and the Research & Development Committee at the (blinded) (blinded).

## Results

### Participants

Of 161 eligible Veterans, 24 Veterans with SCI participated in interviews. Participants were predominantly male (*n* = 21), with an average age of 63 years (range 27–82 years); both are consistent with VA SCI demographics. A large majority of participants (*n* = 19) were from SCI spoke sites, while only 5 were from the hub site. Sixteen participants reported using video telehealth at least once in the past. Generally, participants had at least some access based on technology ownership (e.g., owning at least one internet-capable device) and familiarity with the internet (e.g., spending an average of 3 or more hours/week online). Table 1 provides detailed participant demographic information.

**Table 1:** Participant demographics

	Number	Percent
*Total Veterans (n = 24)*		
Men	21	88%
Women	3	13%
Age (mean and SD)	63 (mean)	± 13.5 (SD)
*Rurality*		
Urban	18	75%
Rural	6	25%
*Level of spinal cord injury*		
Tetraplegia	10	42%
Paraplegia	8	33%
Unknown	6	25%
*Used telehealth*		
Yes	16	67%
No	8	33%
*Technology owned (smartphone, tablet, personal computer)*		
1 type	5	21%
2 types	10	42%
3 types	7	29%
None	2	8%
*Internet utilization (hour per week)*		
0	2	8%
< 3	6	25%
3–9	5	21%
10–19	1	4%
20–39	7	29%
40 +	3	13%

### Context

Interviews began approximately 1 year into the COVID-19 pandemic. Due to the rapid transition to telehealth, VA care initially permitted a range of video telehealth platforms, such as Zoom, Doximity, and VVC, and later transitioned fully to VVC. Therefore, participants’ reported experiences varied by platform and timing regarding the degree of integration of video telehealth into clinical care.

Participants reported a variety of experiences with telemental health, but most found it a helpful tool. Among those who had not used telehealth, most reported openness to trying telehealth. We identified 4 themes illustrating perceptions of telemental health for Veterans with SCI, detailed below:

### Theme 1: Telemental health addresses distance and SCI related access challenges

Veterans with SCI reported several challenges when attending in-person appointments, noting that having an SCI often compounded typical barriers that other Veterans faced. While Veterans most directly referred to distance or travel time needed to get to the VA, they also elaborated on the energy and effort needed to dress or prepare for travel, navigating the physical space (e.g., parking, traversing long hallways), or resources (e.g., family member assistance, transportation services, etc.) needed to get to a VA facility.

#### Distance is more than mileage

Participant_83 noted that trips to VA often take most of the day. He described a recent appointment that involved a 7-h round-trip, though he lives only 90 min from his VA. Sharing that “it takes a minimum of two and a half hours transportation, and it can be up to, you know, 6 o’clock in the morning until 1 o’clock in the afternoon, so telehealth definitely, you know—it’s great for me,” highlighting how telemental health significantly improves the burden of travel for him.

Similarly, multiple Veterans noted how activities of daily living can take significantly more time and planning. Describing how preparing to leave the house is “kind of like taking care of a baby,” Participant_83 elaborated how “you can’t just jump in your clothes and run. You gotta do a lot of preparation before you get out of bed and get in your chair.” Tasks that many able-bodied individuals take for granted take much longer for those with SCI. As evidence, Participant_70 explained, “It takes me almost 20 min of time to tie my shoes, or to get them on my feet.”

Others lamented the physical challenges of navigating the VA campus and facilities because “once I get to the hospital, it seems like a five-mile walk from where I go in to my doctor” (Participant_135). The physical buildings themselves do not seem disability friendly. Even with recent renovations, Participant_70 shared frustrations with long hallways and “steps that’s like full four stories high. How you gonna walk up? I’m in a walker or a wheelchair when I go to the VA. I can’t clear no steps… the place is not Veteran-friendly.” These issues underscore Veterans’ frustrations with access challenges and exemplify how difficult it can be to navigate the world with a disability.

#### Access challenges obstruct the therapeutic process

Pain and frustration related to travel were reported as barriers to productive clinical visits. For example, Veterans who sought treatment for things like PTSD or anger management struggled to absorb and integrate skills when they were already aggravated by simply trying to get to the appointment. Participant_143 shared his “dread” with driving to the VA with “all that traffic, hittin’ every damn light.” Furthermore, the physical activity required to drive and traverse facilities exacerbated pain issues for Participant_113, who struggles with leg spasms and sciatic nerve pain when driving, “it’s real uncomfortable. I sometimes have to stop several times before I get there, just to regroup.” As a result, he drives only when necessary and often arrives at in-person appointments uncomfortable and in a less-than-ideal mental state for therapy.

#### Desire for providers with SCI expertise

Telemental health facilitates access to SCI-competent providers when specialized care is needed. Given the extent of his disabilities, Participant_8 prefers his SCI team because they can provide “specialized care and know my history [so] I don’t have to explain everything over and over again. It helps a lot for me to do the VVC when I can just give updates on what’s going on, rather than having to use both of our time to physically go there.” Relatedly, while many Veterans may live near a VA facility, they may not live near an SCI hub, which can present challenges to accessing SCI expertise for issues related to SCI. In one example, Participant_159 shared that he lived about an hour from a VA clinic but more than 5 h from the nearest SCI hub. The solution was telehealth because it “reduces the travel time … the driving itself is not burdensome by modern standards, but the video telehealth provides a really nice alternative,” underlining the value of telehealth when seeking SCI-informed care.

### Theme 2: Unintended consequences impact access and accessibility of telemental health

Veterans expressed several ways in which processes inadvertently made access more challenging for them.

#### Balancing burden, access, and accessibility

Telehealth applications, such as VVC, were not necessarily designed for people with complex coordination challenges. One Veteran shared that the log-in page asked for name and full address. Typing his information in took so long that the page would time out or fail to save his information when he hit submit. This function was originally designed to support remote providers located far from their patients in case of emergencies during the clinical encounter. However, this increased data-entry burden for those who struggled with low typing proficiency or poor hand coordination.

Other application usability issues included things like the proximity of the buttons. This was the case for Participant_143, who found “it’s too easy to hit the decline and accept. They’re too close together.” Excessive features in the virtual room made the “program that take up even more of the screen. So, you only end up with maybe 40% of the screen is a video picture. It’s better than being on the phone, but it’s still not ideal” (Participant_110).

Participant_50 shared that owning an old smartphone led to her being denied resources. Noting that every time she tried to log into telemental health on her phone, it would crash—“My phone’s not video. Video calls and things like that just drain the battery completely in a very short amount of time.” It took her provider multiple attempts to navigate the VA loaned devices referral process before she was issued a tablet.

#### Creating a therapeutic environment

Another issue frequently referenced was the need to create a virtual therapeutic environment that facilitates rapport and connection. Some issues were general to all telehealth encounters. Veterans often noted issues where a provider failed to set up or use their equipment optimally, which impacted the experience. When asked about the quality of visits, Participant_52 recounted telling his provider, “Your face is on the bottom of the screen, and I keep seeing your chin go below it, and I was more focused on that; it interrupted my thinking.” In another instance, a provider did not have their monitors set up to chart effectively during their session, leading Participant_110 to “see a lot of the back of the doctor’s head and the side of the head because they were busy typing on the computer while we were talking.”

However, some issues specifically impacted accessibility, as was the case for Participant_110, who has hearing loss. He reports that despite having hearing aids, there are still some frequencies and sounds that he cannot hear, so he uses lip reading to augment his hearing capabilities, noting that “some people look away when they’re talking. They’re not talking towards the device. Or not talking very loudly. Or, and this is a big problem for me, when people talk too fast… it sounds more like the Charlie Brown teacher, if you know what I’m talking about.” These issues highlight the importance of simple things, such as maintaining eye contact and facing the patient, to facilitate better experiences.

Even when providers had set up their equipment properly, conducting telemental health visits from shared spaces during the pandemic also required providers to wear masks because they were in close proximity to other staff. Meaning that Veterans with hearing deficits struggled to understand what their provider was saying. As Participant_44 described a recent encounter where his provider “wore a mask, and I had a hard time understanding [them], because I couldn’t watch [their] lips move. And that bothered me, but [they] said [they] couldn’t because there were too many other people in the office.” Masks were mentioned as a common barrier to in-person visits for hearing-impaired Veterans, but they also added frustration during telemental health visits.

Further concerns about shared clinical spaces created concerns about a lack of privacy on a provider’s end as well. Participant_16 shared “my nurse just started doing VVC for the patients that [they] can do VVC with…Like it was [their first time using it, so I had to teach [them] how to use it. And [they were] in a situation where [they were] in the background with other people, and I think everybody can hear me, which I didn’t mind, but I don’t know how much of that is HIPAA violation. We didn’t really talk about much.” Demonstrating the potential impact of conducting telemental health appointments from shared clinical spaces on therapeutic rapport and how that might limit what is disclosed.

However, these problems with creating a therapeutic environment were not limited to one-on-one telemental health encounters. Veterans who attended telemental health groups also expressed dismay when group encounters were not well-moderated to prevent these issues. Participant_57 shared an experience where “a Veteran who was eating, hitting his fork against his plate, making all sorts of noise, you know, and that was very distracting,” demonstrating the value of effective moderation when facilitating a group virtual therapeutic environment.

#### Adaptive equipment facilitates accessibility

Adaptive equipment, backup planning, and support, such as assistance getting started with telemental health, were also important resources in facilitating positive experiences. Equipment ranged from basic solutions like tablet and phone holders to ensure the camera and screen were at a natural viewing angle to more specialized devices like a voice amplification system and voice-activated devices for individuals with higher-level SCIs and more functional impairment. Participant_143 remarked how he has integrated several adaptive tools when using telemental health:

“Voice activation helps me make phone calls on my phone. I got a typing apparatus for my computer when I need to search for something, and it’s not picking up my voice correctly.”

While Participant_37 discussed his use of informal adaptive supports like AirPod headphones to help facilitate his telemental health visits—“My computer’s not loud enough. I have some problems hearing, so I use [AirPods] as a hearing aid.” Meanwhile, other participants discussed needed solutions to address physical challenges, such as when Participant_50 shared “there are times when my hand does this thing where, sort of like the muscle spasms get so bad where my hands just start like, knotting, like just—oh, gosh, sort of like claws,” while describing a desire for an adaptive solution, like a palm grip stylus, suggesting that there may be unmet adaptive needs within this group.

### Theme 3: Adaptive strategies may mitigate telemental health barriers

Generally, Veterans were able to access their telemental health appointments with ease. However, many Veterans reported initial barriers like connectivity, equipment needs, hand dexterity and coordination, and screen size. Table 2 highlights categories of access and accessibility barriers identified in these interviews and provides Veteran- and team-generated solutions used in the clinical resources for providers developed for this project.

**Table 2:** Barriers to telehealth use among Veterans with SCI

Barrier	Illustrative quotes	Solutions
*Connectivity issues*		
Weak or inconsistent Wi-Fi	“Sometimes we can log on, sometimes we can’t… halfway through the call, it will start breaking up… just when we get to the good part… then it drops altogether… so we just revert to a telephone call.” (Participant_14)	Hardwire into internet modem, check download and upload speeds VA Digital Divide Consult Establish a backup plan
SIM card coverage issues on VA-issued devices	“I had to return the first iPad… the second one is hit and miss… there’s not a tower for that provider in my area… so that is what we think is going on.” (Participant_50)	Provider should ask about strongest cellular carrier signal in Veteran’s local area. Be specific in Digital Divide consult notes, so telehealth coordinator can send the best option for Veteran
Delayed technical support	“They don’t get right back to you… normally three days… by the time they called back, I had it fixed.” (Participant_16)	Encourage Veteran to participate in practice calls prior to first appointment Include Initial VVC Visit instructions in the appointment notification Establish a backup plan Have helpdesk number saved in an easily accessible location Collect caregiver information to connect on day of appointment if Veteran is unfamiliar or uneasy with technology
*Equipment issues*		
Resource guarding by providers	“If I’d asked for an iPad, he would have denied it outright… like the money is coming out of his pocket.” (Participant_50)	Educate providers and Veterans on available solutions and benefits of connectivity
Delivery failures/delays	“It was supposed to come to my house… it wound up going to the VA… we lost over a month.” (Participant_113)	Communicate expected timelines to the Veteran so they know when to follow up
*Application issues*		
VA network limitations/lack of platform options	“The VA needs other platforms… Zoom or Messenger… I never have a problem on those… The VA is behind…” (Participant_14)	Emphasize constant quality improvement. VA program offices host meetings and workgroups to gather feedback from the field to make improvements in the preferred platform Consider alternative solutions while awaiting needed improvements
*Dexterity and motor issues*		
Difficulty with login/task workflows	“Logging on… that form you have to fill out… I wish it were streamlined… click the link, click yes, connect.” (Participant_8)	Use speech-to-text where possible Bypass non-essential data entry items Provider enters in name/location info in platform for patients with motor impairment
Neuropathy and reduced fine motor control	“My hands… pins and needles… I drop things… muscles spasm and my hands start like claws.” (Participant_50)	Consider adaptive equipment needs; refer to SCI team or local occupational therapy for recommendations Larger screens may also improve accessibility (be specific in consult)
*Vision issues*		
Small screens	“Not everybody has a laptop… on a phone the small screen is a barrier… not everything displays full screen.” (Participant_16)	VA Digital Divide consult can be placed if patient owned equipment is insufficient. Be sure to document specific issues and needs when applicable Consider Low Vision Clinic referral if appropriate Use large electronic text handouts where possible, Plan additional time to verbally review written information
*Hearing issues*		
Hearing impairment affecting comprehension	“Voice quality… not that good… I have hearing loss… when people talk too fast… it sounds like the Charlie Brown teacher.” (Participant_110)	If using hearing aids, have patient Bluetooth directly to the device to reduce sound distortion Consider using headphones with a microphone rather than open air microphones Eliminate background noise Lower vocal tones, relaxed pace, pause regularly Prioritize eye-contact and verbal teach back methods to confirm understanding

Veterans also reported interpersonal barriers, such as frustration and anxiety that sometimes made it difficult to engage in telemental healthcare. One Veteran spoke at length about anger issues and how he had to develop strategies to build frustration tolerance. Others referred to anxiety exacerbations when they encountered unexpected issues and the different approaches they used. Table 3 provides exemplars of Veteran interpersonal barriers and Veteran-generated solutions to overcome these barriers when using telemental health.

**Table 3:** Strategies Veterans with SCI used to interpersonal barriers like frustration and anxiety

Theme	Illustrative quotes
Using humor & patience to cope with frustration	*“Patience… I get really frustrated. Little mistakes start accumulating… I just want to throw it across the room…I’m starting to see the humor and throw patience at it… it’s slow progress.”* (Participant_52) *“It takes patience on both sides… Not everybody’s 100% on technology… All I can see is opportunity.”* (Participant_110)
Bypassing non-essential steps	*“I filled [the form] all out… had to back out… then I went back… Dang it, I don’t want to fill this out again… realized I didn’t have to fill that stupid thing out…Now, I just kind of ignore the info… you don’t have to fill it out.”* (Participant_8)
Keeping technology updated	*“My main thing… make sure your computer is up to date. Make sure you have the latest browser update… I did the basic troubleshooting… one by one.”* (Participant_16)
Having a backup plan (phone as fallback)	*“If anything messes up, I just say: Hey, we’ll go to the phone.”* (Participant_16)
Requesting materials in alternate formats	*“I couldn’t make [the shared content] out… Normally, I ask them to send me a transcript of what they was showing on the board.”* (Participant_57)
Adapting over time/problem-solving mindset	*“You have to learn a new way to do it… over the years I’ve developed techniques… telehealth presents challenges… I see it as another milestone.”* (Participant_110)

#### Provider knowledge needs, use of resources, and workarounds

One unanticipated challenge that was described by Veterans involved the *perception* of resource guarding among some providers. Veterans shared situations in which they encountered providers who seemed unwilling to refer patients to additional resources, even when the patient directly requested it. Participant_50 described how, when she requested an iPad for telemental health, her provider refused to place a consult and acted like “the money is coming out of [their] pocket, and it’s not.” This did not appear to be a universal issue across all providers in her perception, as she shared a lengthy process in which another provider worked diligently to navigate a complex referral process for the loaned device program until it was successfully approved.

This Veteran also added that sometimes providers were not aware of all the programs and resources available within the VA.

“I’ve had providers, when I ask for something: Well, how’d you learn about this? And sometimes, you know, most times it’s another Veteran, or it’s things that I researched for myself.”

As a workaround to address these issues, she elaborated that Veterans have created an informal network for communicating about resources, which has provided a source of peer connection and support.

“In my experience, and most of the Veterans that I know through the VA, we learn about things by passing on information that we learn, you know, just happenstance, to each other.”

### Theme 4: Flexible use of telehealth can match care modalities to individual needs

Veterans emphasized the importance of flexibility and autonomy in choosing care modalities that meet their situational, emotional, and logistical needs. While interviewers probed for MH perspectives, many Veterans discussed telehealth in the broader context of care, referencing MH and medical care fluidly throughout their conversations.

#### Balancing connection and convenience- benefits of telehealth

Beyond alleviating travel logistics, Veterans commonly cited that telehealth appointments seemed more efficient for the entire healthcare system. Waiting on a provider who is running behind is less burdensome when the patient is at home, as with Participant_109, who”didn’t really care about them being a couple of minutes late because I was still at my house… I just log [onto] Zoom, answer the questions, and log back off, and do what I was doing here.” Related to efficiency for the clinical team, Participant_8 observed how in-person visits require staff to check patients in, nurses to take vitals, and staff to clean/turn over rooms when a patient is done, noting that “it’s burning more time for everybody,” highlighting benefits on both sides of the clinical encounter.

Veterans also valued telehealth for quicker access to scheduling care, troubleshooting, and triaging issues, particularly those who were transit-dependent. Although most examples for troubleshooting and triaging were related to medical issues (e.g., catheter issues, blood pressure, or pain management problems), using telehealth for ad hoc check ins was an important tool, because Veterans felt that having quick and easy access to a care provider went a long way in reducing their anxiety, which is especially crucial for individuals with complex medical needs. Participant_144, highlighting that telehealth can allay “some of your anxiety, your fears of something going wrong where you can actually talk to somebody, either a nurse, PA, or somebody who has some idea,” stressing the value of speedy access.

Telemental health also facilitated connection for Veterans, as many reported that the video option helped promote rapport with their clinical team. Participant_143 shared how telemental health “made it easier for me to relax [and] talk to my psychiatrist. She likes it because I was more open in my house, talking to her, than I would be inside the office, where I’m sure that I don’t know if they can hear me through the doors or not.” In this scenario, telemental health benefited both patient and provider.

Meanwhile, telemental health was used to facilitate connection between Veterans through group Whole Health (a VA program that provides holistic and complementary health programs like Tai Chi) experiences and MH treatment groups. This was especially valuable for individuals who endorsed symptoms of PTSD and challenges with leaving home like Participant_50 who shared that telemental health was great for her needs given that she is prone to “isolate, so you know, doing things through the VA helped with socialization. I slowly became friends with some people there,” demonstrating the value of group telemental health options for this population.

#### ‘It’s not the same, but it comes close’- exploring in person preference

Veterans often expressed a preference for face-to-face appointments while noting the value of using telehealth in certain situations. Some Veterans endorsed feeling more connected to their therapist when they met in person. Participant_8 shared feeling “like there definitely is more of a connection when you’re speaking with the therapist in-person.” However, Veterans who preferred face-to-face appointments also emphasized the value of seeing their provider on video rather than just talking on the phone when in-person appointments were not feasible. Participant_44 remarked that telemental health was “not exactly like being there in person, it’s just not the same. But it is the most acceptable substitute we can find”, while Participant_113 shared how video added to the therapeutic rapport, “I can see the person I’m talking with. You know, it has a more human feel, you know. You can see the person that you’re talking with.” Participant_16 noted the benefits to engagement, especially how providers “can see my expressions and I can see their expressions… Bottom line, it’s more engaging seeing someone.”

Some Veterans expressed the benefits of attending clinical visits in person, with one Veteran endorsing that he was more focused and goal-directed during his in-person visits than during telemental health visits. Participant_52 suggested, “I have more questions after I get off video, at home, or whatever. Maybe in the office I feel more orientated to my—what I’m there for, you know, medical reasons.” Thus, the formality of an in-office visit may facilitate Veterans’ preparedness and engagement.

#### Preferences are not fixed for most Veterans-considering in person and telehealth

When comparing in-person visits to telehealth visits, Veterans noted that face-to-face visits were essential for diagnostic tests, physical assessments, and even when getting to know a new MH provider. However, preferences for telemental health were not static even among Veterans who endorsed in-person care. Veterans shared that when they were struggling with MH or physical symptoms, telemental health made it easier for them to keep appointments and connect with others. Without it, they may have canceled or rescheduled to avoid adding additional stressors of traveling to the VA.

Participant_144 felt telemental health and in-person care worked in parallel, sharing a time his provider used telemental health while he was in distress that resulted in him being willing to re-engage in MH care:

“What you don’t know is what can upset you. And by calming anxiety, calming fears, [I was like] okay, okay, I can—yeah, I think I’ll come in. Yeah, let’s schedule an appointment next week.”

While Participant_50 shared how telehealth allows her to still attend appointments even when her medical symptoms fluctuate:

“If I’m just not feeling well, I can stay home and have my appointment, and I enjoy that. But then there are days when it’s like: Okay, I can go and actually see, you know, it’d be nice to see Dr. [name] or whoever in-person.”

Among Veterans without prior telemental health experience, there was openness to trying it. Some noted a lack of familiarity with technology as a potential barrier, like Participant_70, who heard about telehealth but did not know what it entailed: “I’ve heard it mentioned from time to time, but I didn’t know exactly what it was… I’m not what you call completely computer literate.” Others noted discomfort with providers entering their space. For example, Participant_113 shared how he initially was not interested because he “felt like it was prying [or] getting into my personal space,” but after trying it, he saw “where it can be beneficial for both parties.” However, most Veterans who had not used telemental health attributed their lack of exposure to not knowing it was an option, suggesting that providers are a significant driver of telehealth utilization. Commenting that she “wasn’t asked,” Participant_124 wished, “When the time the virus hit, I could have had some telehealth,” illustrating the powerful role providers play in driving telehealth initiation.

## Discussion

In this qualitative study, Veterans with SCI described telehealth as an important but imperfect tool that enhances access to necessary MH care while reducing logistical barriers that often disrupt continuity, which is consistent with prior literature (Jacobs et al., 2019). For individuals with SCI, lower household income, poorer diet, and lower physical activity levels are associated with poorer mental health, underscoring the value of integrated behavioral health approaches for this population (Sanguinetti et al., 2022). Our findings emphasize the role of telehealth in supporting self-efficacy and health behaviors, aligning with previous research (Jacobs et al., 2019; Kraus et al., 2025; Mirbaha et al., 2023).

Many participants reported that telemental health reduced logistical barriers that frequently disrupt continuity of care, particularly the time, energy, and financial costs, as well as the pain and complex self-care routines required for in-person visits (Mirbaha et al., 2023). For individuals managing the daily routines of SCI, even routine travel to medical appointments can require extensive preparation and physical effort. Some participants reported that, at times, these challenges deter them from attending in-person appointments, especially when the difficulties outweigh the perceived benefit. In these circumstances, telemental health reduced these barriers by offering a realistic, lower-threshold means to engage in care, supporting self-efficacy and agency in directing one’s own treatment (Anderson et al., 2022). Participants also highlighted that having a choice between modalities (i.e., in-person vs telehealth) was important for remaining engaged in treatment.

Across interviews, Veterans emphasized the particular importance of working with providers who understand the realities of living with SCI. Within the VA’s hub and spoke system for SCI care, some Veterans live far from specialized SCI centers and may otherwise receive care from providers with less experience managing SCI-related needs. In these situations, telehealth enables continued access to trusted clinicians who were familiar with their medical histories and the psychosocial implications of living with SCI (Daly et al., 2025; LaVela et al., 2004). The current literature indicates a growing shortage of SCI providers in training each year; as a result, the VA has the most comprehensive system of care for this disorder (Svircev et al., 2022). Additionally, evidence suggests that Veterans using community care for mental health services report lower satisfaction with community care than VA services, reinforcing the vital importance of VA’s comprehensive system of care in both SCI and MH domains.

Importantly, many Veterans without previous telehealth use indicated that lack of awareness, not lack of interest, was the primary barrier. Several Veterans reported that they had never been offered video visits or did not understand how telehealth worked. This varied based on the timing of the interviews and the early COVID-19 rapid transition to telehealth. However, this experience underscores the value of retaining telehealth practices in routine care to ensure access and accessibility for the SCI population during unexpected challenges.

Veterans also shared several access and accessibility issues that impeded a smooth clinical experience during telemental health sessions (e.g., applications timing out due to poor hand dexterity and slow typing, applications blocking voice-to-text, etc.). Although many of these issues were not unique to the SCI experience, solutions sometimes required adaptive equipment, alternative approaches, or specialty knowledge to effectively navigate system supports and accessibility issues. These findings support the value of applying universal design principles when developing new systems, processes, and applications to ensure accessibility is integrated as a standard rather than an exception (Annaswamy et al., 2020; Kobeissi & Manning, 2025; Touchett et al., 2022; Valdez et al., 2021).

There were ample examples of challenges in establishing an effective therapeutic environment during video visits, which likely reflect the impact of the rapid transition to telehealth without comprehensive training. For example, Veterans noted that provider behaviors, such as looking away from the camera, speaking while facing another monitor, or conducting visits in shared clinical spaces, could disrupt rapport and make communication more difficult. These issues made telemental health visits particularly challenging for Veterans with hearing impairment, who may rely on visual cues such as lip-reading. In some cases, providers wearing masks while conducting video visits further limited communication. These challenges shared by Veterans with SCI suggest that the effectiveness of telemental health is influenced not only by the availability and accessibility of technology, but also by how clinicians use it during telehealth visits (Lindsay et al., 2021). Simple practices such as maintaining eye contact with the camera, minimizing distractions, ensuring adequate lighting and sound, and conducting visits from private spaces may improve the patient experience (Koppel et al., 2025; Touchett et al., 2022).

Veterans consistently emphasized the value of flexibility in care modalities, noting that in certain situations, they preferred in-person appointments. For instance, Veterans stated that they felt more connected to their therapist when they met in person and noted that specific types of visits necessitated an in-person appointment, such as diagnostic tests, physical assessments, and establishing rapport with a new provider. Rather than viewing telehealth as a substitute for in-person care, Veterans described it as one option within a broader continuum of care that can be matched to situational needs. These findings support that maintaining flexibility in care delivery models may be particularly important for individuals with complex disabilities (Tan et al., 2025).

Our results support existing literature that provider recommendation, engagement, and patient education play a critical role in telehealth adoption (Kobeissi & Manning, 2025; Martinez et al., 2017; Valdez et al., 2021). Providing clear information about telehealth options may allow Veterans to make informed choices about how they access care. One of the many ways we have coached providers to approach Veterans about telemental health is to use a few minutes of an in-person appointment to show a Veteran the telehealth platform, so the Veteran can understand the benefits and limitations of telemental health, and how to access it. Taken together, these findings suggest that telehealth holds promise for improving access to MH care for Veterans with SCI, but its effectiveness depends on the thoughtful implementation (Touchett et al., 2022). Telehealth programs that are not designed with disability accessibility in mind risk creating new barriers even as they attempt to remove existing ones (Phuong et al., 2023). Designing telehealth platforms with accessibility features, ensuring availability of adaptive equipment, and educating providers on disability concepts and best practices for virtual therapeutic environments may substantially improve the experience for patients with complex disabilities (Kobeissi & Manning, 2025). Integrating these modalities into usual care will also support a seamless transition in future times of need.

### Limitations and future directions

This study has several limitations that should be considered. First, the sample may reflect self-selection, as individuals strongly opposed to telehealth may have been reluctant to participate. In fact, one eligible participant declined the interview on this basis. As a result, participants may have been more open to telehealth than the broader population of Veterans with SCI. Future work should intentionally broaden recruitment and include comparative groups to better understand differences in telemental health engagement and to enhance the transferability of our findings.

Second, data were collected between 2021 and 2022 during the later stages of the COVID-19 pandemic, a period characterized by the rapid expansion of telehealth services. Telehealth infrastructure, provider experience, and patient familiarity with video visits have continued to evolve since that time. As a result, some barriers described by participants may have changed as telehealth systems improved. However, these insights may guide ongoing evaluations and process improvements.

To ensure telehealth serves as a facilitator and not an added barrier, future efforts should prioritize research and program development accessibility from the outset, expanding telemental health options that meet the complex medical and functional needs of individuals with SCI, as demonstrated in emerging teleSCI models and innovative virtual care approaches (Touchett et al., 2022). Strategies should focus on adapting and extending existing evidence-based interventions rather than independently recreating solutions to ensure alignment with patient needs and health system capabilities (Touchett et al., 2024).

The prevalence of MH issues among individuals with SCI is not unique to those with Veteran status (Peterson et al., 2022). Given ongoing specialty and provider shortages in rural and medically underserved areas (Arredondo et al., 2023; Svircev et al., 2022), telemental health holds substantial promise for improving access to high-quality SCI care across geographic settings, underscoring the importance of continued evaluation and development in this space.

Policy makers should prioritize: a) the development of a standardized process to provide adaptive equipment for the Veterans that need it, b) provider education in disability competent telemental healthcare, c) adaptable telehealth workflows to ensure reliable connectivity support, scheduling flexibility, and clear options to switch between modalities, and d) routine integration of video-based care since lack of awareness was a primary reason some had not used telehealth.

## Conclusions

For Veterans with SCI, telemental health is more than a convenience; it is a behavioral medicine strategy that has the potential to strengthen engagement, autonomy, and continuity of care in the face of compounding structural and disability-related barriers. When implemented thoughtfully, telehealth can reduce logistical barriers, support continuity of care, and facilitate access to specialized providers. However, maximizing its benefits requires attention to access, accessibility, disability-competent care, and the creation of effective virtual therapeutic environments. Addressing these factors may allow telehealth to better support MH care engagement for Veterans living with complex disabilities.

## Data Availability

The data that support the findings of this study are not openly available due to reasons of sensitivity and are available from the corresponding author upon reasonable request.
